# Migrant workers with COVID-19: Recognizing the crucial role non-governmental organizations perform

**DOI:** 10.1016/j.lanwpc.2021.100145

**Published:** 2021-04-13

**Authors:** Bingwen Eugene Fan

**Affiliations:** a Department of Haematology, Tan Tock Seng Hospital, Singapore; b Department of Laboratory Medicine, Khoo Teck Puat Hospital, Singapore; c Lee Kong Chian School of Medicine, Nanyang Technological University, Singapore; d Yong Loo Lin School of Medicine, National University of Singapore, Singapore

**Keywords:** COVID-19, Migrant workers, Non-governmental organizations, Mental health

I read with interest “Building community resilience beyond COVID-19: The Singapore way” [1] where various government-led policies and measures in mitigating the spread of COVID-19 have been excellently outlined. However, the discussion of community efforts to combat COVID-19 could be more balanced by highlighting the crucial role that non-governmental organizations (NGOs) have performed in establishing ground-up initiatives for our migrant workers.

As of 18^th^ March 2021, the number of migrant workers with COVID-19 stands disproportionately at 54,511 cases (90.6%) out of a total of 60,152 cases [2], in contrast to most countries where citizens are mostly affected. With dormitory outbreaks of COVID-19 in April 2020, measures were taken for mass quarantine and mass testing of migrant workers. While the inter-agency task force moved quickly to address the needs of migrant workers in big dormitories, several non-governmental organizations such as Healthserve, Transient Workers Count Too, Alliance of Guest Workers Outreach, COVID-19 Migrant Support Coalition, Crisis Relief Alliance, Migrant Workers’ Centre and It's Raining Raincoats rose to the challenge of meeting the basic needs (food, toiletries) of migrant workers confined in the 1,100 smaller housing facilities (housing 30-900 workers) [3] and disseminating information on physical distancing and personal hygiene. Healthserve also focused on key mental and psychosocial aspects [4], including: 1) Partnering of on-site medical teams to conduct small group needs assessment, addressing concerns such as fears of COVID-19 transmission and deportation 2) Setting up a multilingual COVID-19 information webpage which addressed mental health and psychosocial concerns 3) Partnership with the National Centre for Infectious Diseases (where majority of COVID-19 cases were treated) to create videos and patient information leaflets in native language to explain hospitalization and isolation processes, booths for online remittance and even prayer mats for Muslims wishing to observe Ramadan 4) Supporting migrant workers through a crisis hotline and web-based Tele-Befriending virtual clinic with trained volunteers and volunteer psychiatrists 5) Crowdfunding campaigns for deceased or debilitated migrant workers from COVID-19 through platforms such as Give.Asia. These broad measures demonstrate how NGOs can engage with government agencies to ensure accessible services, balanced policies, and equity for migrant workers [5].

Globally, migrant workers remain a vulnerable group, encountering barriers to equal access to healthcare, resources and vaccinations as compared with the host country's citizens. Despite COVID-19 further widening the preexisting fault-lines of inequality, a silver lining emerges: the humanity, compassion, and generosity toward the less fortunate from a concerned citizenry through NGOs.

## Declaration of Competing Interest

I have none to declare.

## References

[1] Yip W, Ge L, Ho AHY, Heng BH, Tan WS. (2021). Building community resilience beyond COVID-19: the Singapore way. Lancet Reg Health West Pac.

[2] COVID-19 situation report (Data Updated as of: 18 Mar 2021) ministry of health, Singapore website. https://covidsitrep.moh.gov.sg/

[3] (2020). Coronavirus: NGOs step in to help migrant workers at smaller housing facilities. https://www.straitstimes.com/singapore/coronavirus-ngos-step-in-to-help-migrant-workers-at-smaller-housing-facilities.

[4] Chan LG, Kuan B. (2020). Mental health and holistic care of migrant workers in Singapore during the COVID-19 pandemic. J Glob Health.

[5] (2020). Preparedness, prevention and control of coronavirus disease (COVID-19) for refugees and migrants in non-camp settings: interim guidance.

